# Reflex neurosis (NEAD)

**DOI:** 10.4103/0019-5545.39767

**Published:** 2008

**Authors:** V. A. P. Ghorpade

**Affiliations:** Department of Psychiatry, M.S. Ramaiah Medical College, Bangalore, Karnataka, India. E-mail: anandoparkashg@yahoo.com

Sir,

Provoking an epileptic attack by pouring of hot water on the head is recognized as “HOT WATER EPILEPSY” (HWE), which is now considered as a form of reflex epilepsy. Venkataramiah[1] observed that most of these patients had a phobia for the process of bathing. Here I would like to reinforce this point with a case report and its implications in practical management of such cases in one's practice.

A 17-year-old girl was referred to the present author as a case of hot water epilepsy with severe phobia for hot water head bath, for psychological treatment by a neurologist colleague after his neurological evaluation revealed no Electro Encephalographiy (EEG) and Radiological (X-ray of the skull) abnormalities.

Psychiatric evaluation revealed that at the age of 13, she used to sit down exhausted after having a hot water head bath. Later, she used to lose consciousness gradually after every hot water head bath regularly. No motor seizure or aura was reported during these periods. Three months before the consultation, she started complaining of buzzing sound in the ears, and past memories would flash in her mind whenever she heard the sound of mixing water for bath, heard the sound of someone taking bath or when she desired to take a head bath. This was accompanied with muttering, staring look, automatisms of lips, and amnesia. Later she started getting such attacks even with hot water body bath; these never used to occur with cold water bath. As a result, she used to take body bath or head bath only in the presence of a family member, who waited outside the bathroom, or with the bathroom door kept half open. When family members were not present, she used to take bath as quickly as possible and come out of the bathroom. These symptoms started troubling her even when she thought about bath or heard the sound of bathing. As a result, she started avoiding bathing as much as possible. Past history, family history, and findings of physical examination were not contributory. Mental status examination revealed phobia of hot water head bath.

A provisional diagnosis of phobic neurosis of hot water head bath with HWE? was made and was managed on the principles of desensitization. Behavior treatment consisted of motivating her to take bath with hot water of different temperatures, in ascending order. At that time, the clinician and the relative use to be present nearby, so that patient could regain the confidence to take bath independently. This was repeated daily till the patient was able to take bath without any difficulty or did not develop any “seizure.” This was followed with regular supportive psychotherapy. This approach yielded encouraging results. When the patient reported being fully confident to take bath independently and enjoyed it and symptoms did not recur, the treatment was terminated. At the end of the treatment, bathing failed to provoke any symptoms. One month later, she reported that she was enjoying the bath and was completely relieved of all the symptoms with which she had presented. No antiepileptic drugs were prescribed.

Various neuropsychiatric symptoms following hot water head bath were observed by Subramanyam HS[2]; there was no mention of cases with phobia of hot water head bath, indicating the rarity of this condition. It is not necessary that all neuropsychiatric symptoms provoked by hot water head bath are epileptic in nature, as Schmidt[3] reports that 15-30% of the cases referred to a specialized epilepsy center did not suffer from epilepsy. Mani *et al.*[4] report that 13% of seizure suspects in a survey did not have epilepsy, and Nag[5] reports that 17-28% of patients are on antiepileptic drugs even though they do not have seizures. What could be the nature of these cases which were not epileptic but developed neuropsychiatric symptoms following hot water head bath? This case report suggests that some or all of them could have neuropsychiatric symptoms arising out of psychological factors.

Antebi and Bird[6] in their excellent review of reflex epilepsy have expressed the following opinion: “Not everyone who has seizure has epilepsy. There is also the inevitable contamination of non-epileptic phenomena or pseudo seizures; and no matter how stringent the diagnostic criteria, a degree of it is inevitable.”

Diagnostic dilemma between genuine seizure and pseudo seizure could be resolved by provoking the “seizure” with the stimuli, viz., hot water head bath, with simultaneous recording of EEG and ECG,[7] which may not be practical and perfect as highlighted by Mani *et al.*[4] and Subramanyam.[2]

Venkataramiah[1] and the present author used the method of provoking a seizure with hot water head bath as a diagnostic and therapeutic tool. This simple approach can be used when hi-tech investigations are not available; consequently, unwanted long-term anti-epileptic medications can be avoided, keeping in mind the increased medicolegal awareness in the society.

As HWE is considered a variant of reflex epilepsy, similarly, varied behavior (some can have just breath-holding attack, others can lose consciousness, and some others may develop automatisms) provoked with hot water head bath can be labeled “reflex neurosis.” The current trend is to use the term NEAD (non-epileptic attack disorder) for such varied picture.[8]

This simple procedure that has been adopted here can be adopted as a treatment procedure for those who have phobia with or without epileptic component. More controlled studies can clear the darkness prevailing on this disorder.
